# A Novel Homozygous Mutation of Thyroid Peroxidase Gene Abolishes a Disulfide Bond Leading to Congenital Hypothyroidism

**DOI:** 10.1155/2020/9132372

**Published:** 2020-08-30

**Authors:** Fumiyoshi Yakou, Hirotsugu Suwanai, Takuya Ishikawa, Mariko Itou, Jumpei Shikuma, Takashi Miwa, Hiroyuki Sakai, Kohsuke Kanekura, Satoshi Narumi, Ryo Suzuki, Masato Odawara

**Affiliations:** ^1^Tokyo Medical University, Department of Diabetes, Metabolism and Endocrinology, Tokyo 160-0023, Japan; ^2^Tokyo Medical University, Department of Molecular Pathology, Tokyo 160-8402, Japan; ^3^Department of Molecular Endocrinology, National Research Institute for Child Health and Development, Tokyo 157-8535, Japan

## Abstract

Congenital hypothyroidism (CH) is the most prevalent congenital endocrine disorder and causes mental retardation. A male Japanese patient with first cousin marriage parents was diagnosed as CH at 10 months. He was born before introduction of mass screening for CH. With continuous thyroid hormone replacement therapy, normal thyroid hormone status was maintained until adulthood. Genetic screening of next-generation sequencing was performed at the age of 52 years, and we identified a new homozygous thyroid peroxidase (TPO) gene mutation (GRCh38.p13, chromosome 2 at position 1493997, c.1964 G>T, p.Cys655Phe). TPO is an important enzyme to produce thyroid hormone. As demonstrated by a homology analysis of TPO proteins among different species, cysteine 655 residue is highly conserved, suggesting an important role in maintaining TPO function and structure. An *in silico* study with three-dimensional structure of the novel mutation was performed and suggested that the mutation abolished disulfide bond between cysteines at positions 598 and 655. An *in vitro* functional analysis using HEK293 cells revealed that TPO activity of the mutant was significantly impaired compared with that of the wild type. Furthermore, study of immunohistochemistry showed that localization of TPO in cells did not differ between the wild type and the mutant. In conclusion, this single disulfide bond loss mutation of a new TPO homozygous mutation, p.Cys655Phe, reduced TPO activity and caused congenital hypothyroidism without affecting subcellular localization of TPO proteins.

## 1. Introduction

Congenital hypothyroidism (CH) is the most prevalent congenital endocrine disorder and one of the preventable causes of mental retardation [1]. Prevalence of CH is approximately 1 in 2,000–4,000 newborns all over the world [2]. In Japan, mass screening for CH was introduced in 1979 and is usually performed in the neonatal period. Genetic screening of CH has been performed for research purpose and mainly eleven genes are related to CH such as *TSHR*, *PAX8*, *NKX2-1*, *FOXE1*, *TG*, *TPO*, *SLC5A5*, *SLC26A4*, *IYD*, *DUOX2*, and *DUOXA2*. Thyroid dysgenesis accounts for 80–85% cases, while 10 to 20% cases of CH are due to abnormalities in thyroid hormone synthesis [3]. Thyroid peroxidase (TPO) deficiency due to a biallelic *TPO* mutation is a representative genotype of CH [4]. Inheritance pattern of CH due to *TPO* mutation is autosomal recessive. Most patients with biallelic *TPO* mutations exhibit permanent CH.

TPO plays essential roles in thyroid hormone production. Oxidized iodide by TPO attaches to tyrosyl residues in thyroglobulin (Tg) to make monoiodotyrosine and diiodotyrosine, a process also catalyzed by TPO. Next, these iodotyrosyl residues couple in another TPO-mediated reaction to form an iodothyronine, triiodothyronine (T3), or thyroxine (T4) [5]. Therefore, individuals with low TPO activity may have insufficient thyroid hormone synthesis.

Here, we report a new homozygous *TPO* mutation (GRCh38.p13, chromosome 2 at position 1493997, c.1964 G>T, p.Cys655Phe) identified via genetic screening based on next-generation sequencing. To date, approximately 70 *TPO* mutations have been recorded in the Human Gene Mutation Database (http://www.hgmd.cf.ac.uk/ac/index.php). However, this is an unrecorded and novel mutation. Therefore, we performed conformational prediction and *in vitro* analyses of the novel *TPO* mutation in this case.

## 2. Materials and Methods

### 2.1. Patient

This study was approved by Tokyo Medical University, medical ethics committee (SH2932). Written informed consents were obtained from the proband and his elder brother. A male patient, the fourth child of healthy Japanese consanguineous parents, was born at term after an uneventful pregnancy and delivery. He was born in 1979, before the introduction of mass screening for CH. His family reported that the patient presented persistent drowsiness, could not drink breast milk, and was hospitalized soon after birth. Although he was treated with nutrition therapy, height and weight gains were delayed. He was diagnosed as CH at the age of 10 months based on the blood test, and thyroid hormone replacement therapy was initiated. His thyroid hormone status remained normal since then until now. There were no problems in his growth process, but he has a mild intelligence deficit. His age at the last visit was 52 years. He was 162 cm tall and weighed 58 kg. He has been our outpatient from the age of 37 years and has received thyroid hormone replacement therapy. At present, we continue administering him with levothyroxine at 150 *μ*g/day. In palpation, his thyroid gland was soft, mobile, and symmetric. Recently, his hormone levels associated with thyroid were within the normal range at the last visit: serum TSH 1.04 *μ*U/mL (reference 0.46–3.50) and free T4 1.50 ng/dL (reference 0.90–1.80). Serum Tg values during the visit term varied between 59.0 and 546.0 ng/ml (reference <32.7) in a few measurements. Serum levels of anti-Tg antibody and anti-TPO antibody were in normal range.

### 2.2. Detection and In Silico Analysis of TPO Mutation

Peripheral venous blood samples were obtained from the proband and his elder brother. Genomic DNA was extracted from peripheral blood leucocytes using the Gentra Puregene Blood Kit (Qiagen, Germany) according to the manufacturer's protocol. The CH capture panel contained 11 known CH-related genes, 3 of which (*PAX8*, *NKX2-1*, and *FOXE1*) are involved in thyroid dysplasia [4]. TSHR is a hormone receptor involved in TSH signaling abnormalities. The remaining seven genes (*TG*, *TPO*, *SLC5A5*, *SLC26A4*, *IYD*, *DUOX2*, and *DUOXA2*) are involved in thyroid dyshormonogenesis [6].

For gene analysis, next-generation sequencer MiSeq (Illumina Inc, San Diego, CA, USA) was used according to the SureSelect protocol (Agilent Technologies, Santa Clara, CA, USA), as described previously [7]. Using the Illumina processing pipeline, base calling, read filtering, and demultiplexing were performed. BWA 0.7.17 was used for alignment against the human reference genome (NCBI build 37; hg19) [8]. Local realignment, quality score recalibration, and variant calling were performed using GATK 3.8 with default settings [9]. ANNOVAR was used for the annotation of the detected variants [10]. ClustalW (version 2.1) was utilized to perform multiple sequence alignment of *TPO* family proteins from different species [11].

The detected mutation was confirmed using standard polymerase chain reaction- (PCR-) based Sanger sequencing, as described previously [12]. We referred to mutation frequencies in variant databases, including the Genome Aggregation Database (https://gnomad.broadinstitute.org), 1,000 Genomes Project (http://www.internationalgenome.org/), Human Genetic Variation Database (http://www.genome.med.kyoto-u.ac.jp/SnpDB/), and ToMMo 4.7KJPN (https://jmorp.megabank.tohoku.ac.jp/). Descriptions of the mutations were based on Homo sapien*s TPO* (National Center for Biotechnology Information (NCBI), reference sequence: NM_000547.5).

*In silico* studies were performed with the wild type (WT) and mutant *TPO* variants using PyMOL 0.9 to evaluate the three-dimensional (3D) structure of p.Cys655Phe TPO [13]. For the conformational prediction of TPO, human myeloperoxidase (MPO) 3D structure was used as a template. MPO is known as the closest homolog to TPO and shares 47% sequence identity with the MPO-like domain of TPO [14, 15]. The X-ray crystal structure of human MPO has been previously determined (PDB accession code 3F9P) [16].

### 2.3. Cell Culture and Functional Analyses

HEK293 cells were maintained in Dulbecco's modified Eagle's medium that was supplemented with 50 U/mL penicillin, 50 *μ*g/mL streptomycin, and 10% fetal bovine serum. An expression vector encoding C-terminal hemagglutinin-tagged human *TPO* (TPO-HA) was created by inserting the *TPO* cDNA sequence into pEGFP-N1 (Clontech laboratories, Palo Alto, CA) as previously described [17]. After inserting the *TPO* cDNA sequence into pB513B-1 (System Biosciences, Palo Alto, CA, USA), an expression vector for stable TPO expression (WT) was created. A novel *TPO* mutation (p.Cys655Phe) expression vector was created by site-directed mutagenesis. The stable human embryonic kidney 293 (HEK293) cell lines expressing each TPO protein (WT, p.Cys655Phe) was established using the PiggyBac system according to the manufacturer's protocol.

Cell transfection was performed using Lipofectamine 3000 reagent (Invitrogen, Carlsbad, CA, #L3000008). The cells were seeded into 12-well tissue culture plates at a density of 0.1 × 10^6^ cells/well to reach approximately 70%–90% confluence for transfection. Transfection reagent and 1 *μ*g DNA (composed of 750 ng p.Cys655Phe expression vector and 250 ng transposase) were added to 100 *μ*L Opti-MEM medium and placed at room temperature for 5 minutes. Mixtures were added to the seeded cells and were cultured in an incubator at 37°C, 5% CO_2_ for 3 hours. After incubation, the cells were replaced with the conventional medium and selected by puromycin and cultured sequentially for 48 hours. Transfected cells were continuously cultured, as described above, and stable HEK293 cell lines expressing each TPO protein (WT, p.Cys655Phe) were established.

For TPO activity measurement, we prepared 90% confluent stable cells expressing each TPO protein (WT, p.Cys655Phe) in 12-well dishes. Cells were trypsinized, washed with phosphate buffered saline, and resuspended in 1X Earle's balanced salt solution (Sigma Aldrich, St. Louis, MO, USA) containing 100 *μ*M Amplex Red reagent (Thermo Fisher Scientific) and 2 mM H_2_O_2_, as previously described [18]. After transferring to a 96-well plate, the mixtures were incubated at 37°C for 1 hour and stirred frequently. Fluorescent emission was measured using EnSpire™ Alpha (Molecular Devices, PerkinElmer, Inc, Waltham, MA, USA) to quantify TPO activity of the cells. TPO activity of the new mutant is expressed as percentage (mean ± standard error of the mean (SEM)) of the WT activity. The background activity, which was measured using nontransfected cells (control), was set to 0%. The above experiment is representative of procedures performed independently three times and obtained similar results. *P* values <0.05 obtained using Student's *t*-test were considered significant.

### 2.4. Western Blot Analysis

Stable cells were grown in a 100 mm plate to approximately 90% confluency, washed twice with PBS, and lysed using the RIPA Lysis Buffer System (Santa Cruz, Dallas, TX, USA, #sc-24948) protein extraction method. Western blotting was performed using anti-TPO rabbit monoclonal antibodies (Abcam, Cambridge, MA, USA; ab109383) as the primary antibody and KPL peroxidase-labeled antibodies to rabbit IgG (H+L) (SeraCare, Milford, USA; 5220-0336) as the secondary antibody. Furthermore, we used mouse monoclonal anti-beta-actin (Abcam, Cambridge, MA, USA; ab8226) as the primary antibody of the loading control and rabbit anti-mouse IgG H&L (Abcam, Cambridge, MA, USA; ab6728) as the secondary antibody. Each band was analyzed using image processing software ImageJ (http://imagej.nih.gov/ij/), and the expression levels were examined. Reverse transcription of RNA and the real-time PCR were performed according to manufacturer's instructions, using KAPA SYBR FAST quantitative PCR Master Mix Kit (KAPA BIOSYSTEMS, Cape Town, South Africa; KK4601) and quantitative PCR primer pairs for *TPO* (NCBI Reference Sequence: BC095448; forward primer sequence: 5′-AACAACAGAGACCACCCCAGAT-3′, reverse primer sequence: 5′-TGACTGAAGCCGTCCTCATAGAC-3′) or housekeeping gene *hprt1* (NCBI Reference Sequence: NM_000194; forward primer sequence: 5′-CATTATGCTGAGGATTTGGAAAGG-3′, reverse primer sequence: 5′-CTTGAGCACACAGAGGGCTACA-3′). TPO quantitative PCR were shown in arbitrary units of TPO mRNA/hprt1 as the mean ± standard error of the mean.

### 2.5. Immunohistochemistry

HEK293 cells overexpressing wild type or mutant TPO were seeded on a chambered coverglass and then transfected with pDsRed2-ER Vector (Clontech laboratories, Palo Alto, CA, 632409). Twenty-four hours after transfection, the cells were fixed with 10% formalin-phosphate buffer solution (PBS) for 15 minutes at room temperature. After washing three times with PBS, the cells were treated with or without 0.5% Triton-X100 for permeabilization, blocked with BlockingOne (Nacalai tesque, Kyoto, Japan, 03953-66), and incubated with anti-TPO antibody (Abcam, Cambridge, MA, USA; ab109383) for 2 hours at room temperature. After washing four times with PBS, the cells were incubated with Alexa-Fluor488 labeled Goat anti-Rabbit IgG (H+L) secondary antibody (Thermo Scientific, #A32731) for 1 hour at room temperature in a dark room. After washing four times with PBS, the cells were mounted with ProLong Gold with DAPI solution (Thermo Scientific, #3693).

## 3. Results

### 3.1. Mutation Analysis of the TPO Gene

**Figure 1:**
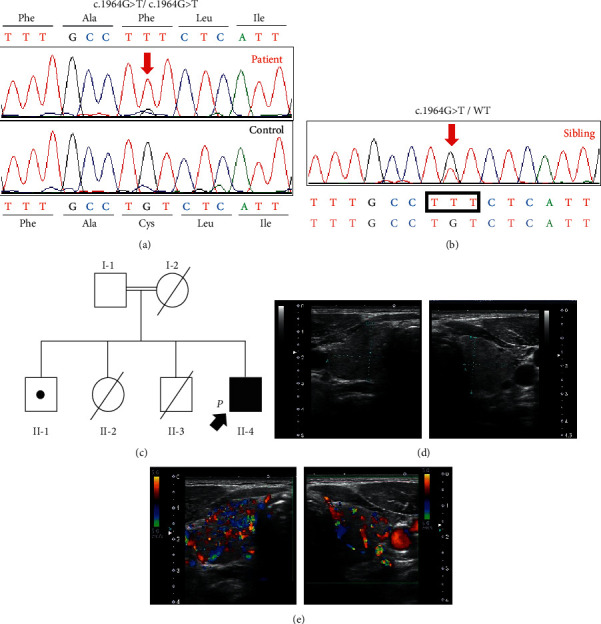
Discernment of a *TPO* mutation. (a) Partially electrophoretic pattern of the discerned TPO mutation. The patient had a homozygous novel missense mutation (p.Cys655Phe, red arrow). (b) Partially electrophoretic pattern of the discerned TPO mutation. Elder brother (Figure 1(c), II-1) had a heterozygous novel missense mutation (p.Cys655Phe, red arrow). (c) Family tree of the patient. Filled symbols with a *P* in the lower left indicate the proband. The dots in the squares indicate carriers. We have not examined the parents and siblings of the proband for mutations. Image inspection result of the patient. (d) Ultrasonography showed mild goiter in the patient. (e) Internal echoes were uneven, and increased blood flow was observed on a color Doppler ultrasound.

**Figure 2:**
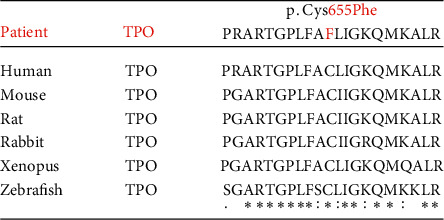
Comparison of amino acid sequences with various mammalian species amino acid sequences containing the novel mutations was compared with those in other mammalian species. ClustalW calculates the best match for selected sequences and lines them up so that identities and differences can be observed. The arrow indicates the position of the amino acid substitution Cys655Phe. The asterisks show perfectly preserved amino acids. The colons show high homology. The dots show low homology. The amino acid sequences are based on Ensembl, which is a genome browser for vertebrate genomes, and NCBI, which houses a series of databases relevant to biotechnology and biomedicine and is an important resource for bioinformatics tools and services. Human *TPO* (ENST00000329066.8), mouse *TPO* (ENSMUST00000021005.14), rat *TPO* (ENSRNOT00000006526.3), rabbit *TPO* (ENSOCUT00000016422.3), xenopus (tropical clawed flag) TPO (ENSXETT00000002202.1), and zebrafish (Danio *rerio*) *TPO* (XP_021322945.1).

Genetic screening of next-generation sequencing for the proband was performed, and a new homozygous TPO gene mutation (GRCh38.p13, chromosome 2 at position 1493997, c.1964 G> T, p.Cys655Phe) was identified. He did not have any mutations in the remaining genes that were analyzed. A homozygous novel TPO mutation was confirmed via Sanger sequencing (Figure 1(a) red arrow; Figure 1(c) II-4). His sibling (Figure 1(b); Figure 1(c), II-1) had the same mutation in the state of heterozygous confirmed by Sanger sequencing. His parents were first cousins (Figure 1(c), I-1 and I-2) and probably carriers of heterozygous TPO mutation. The patient's mother had already died, and his father was too old to visit. Therefore, blood samples of parents could not be collected.

Thyroid ultrasonography showed mildly enlarged thyroid (estimated volume, 22.9 mL; Figure 1(d)), uneven internal echo imaging, and increased internal blood flow (Figure 1(e)). His elder brother (Figure 1(c), II-1) has no goiter and has normal thyroid hormone levels, with no conspicuous abnormalities observed in growth, intelligence, and adolescent development. His elder sister (Figure 1(c), II-2) and second brother (Figure 1(c), II-3) died shortly after birth. Detailed records were not available. According to family information, autopsy of both children revealed enlarged thyroid glands. The family had no history of thyroid cancer.

Homology analyses of protein sequences across species were performed around Cys655 of human TPO proteins using ClustalW 2.1 software. The Cys655 residue substituted with the mutant was highly conserved among mammalian species (Figure 2).

### 3.2. Conformational Prediction

**Figure 3:**
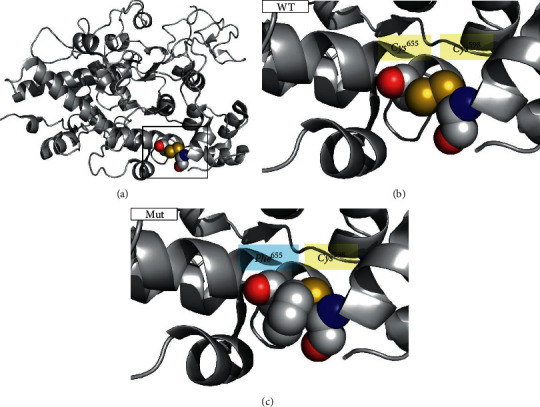
Conformational prediction of novel TPO mutation. (a) Human TPO structure diagram. Figures B and C are enlarged versions. (b) The wild type of Cys655 (WT). (c) The mutant type of Phe655 (Mut). These figures indicate that the substitution of p.Cys655Phe abolishes disulfide bonds between cysteines at positions 598 and 655. The yellow part is sulfur, the red part is nitrogen, the blue part is oxygen, and the gray part is carbon.

As demonstrated by mutation detection, the cysteine 655 residue is within a highly conserved region of TPO, suggesting its important role in TPO function and structure. Cysteines can joint between side chains via disulfide bonds as part of the secondary and tertiary structures of proteins. A comparison of the predicted tertiary structure of the WT and mutant protein revealed that the novel *TPO* mutation p.Cys655Phe abolishes disulfide bonds between cysteines at positions 598 and 655 (Figures 3(a)–3(c)).

### 3.3. Functional Analysis

**Figure 4:**
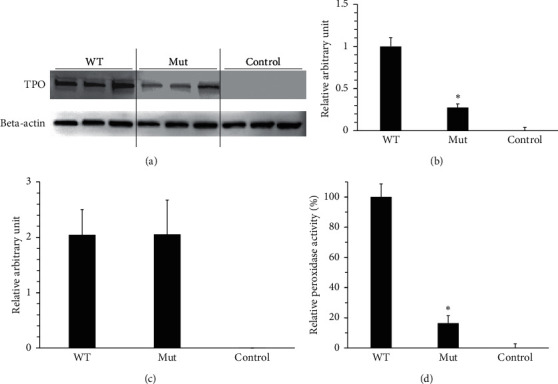
Functional analyses of the identified novel TPO mutation. Protein expression levels of TPO (WT or Mut) were assessed using Western blotting with a monoclonal anti-TPO antibody. (a) The upper bands denote TPO proteins (103 kD). Monoclonal anti-beta-actin antibodies were used as the loading control. The lower bands denote beta-actin proteins (42 kD). Western blots were performed in triplicate. (b) Each TPO expression signal was expressed in relative arbitrary unit after normalizing against beta-actin (TPO/beta-actin ± SEM). Wild-type TPO expression level was set to 1, and control TPO expression level was set to 0. The mutant TPO expression level was significantly lower than that of the wild type (*n* = 3, Student's *t* test, ^*∗*^*P* < 0.05). (c) The results of quantitative PCR performed using SYBR Green were shown in arbitrary units of TPO mRNA/housekeeping gene hprt1 as the mean ± standard error of the mean. There was no significant difference between wild-type and mutant TPO mRNA expression (*n* = 5, Student's *t* test, *P*=0.990). Measurement of peroxidase activity using Amplex Red reagent. (d) Peroxidase activity of mutant TPO protein (Mut) was normalized to that of the wild type (WT; 100%) and that of the mock-transfected (control; 0%). The results of three independent experiments are expressed as the mean ± standard error of the mean. ^*∗*^*P* < 0.05, Student's *t*-test (Mut vs WT).

We performed *in vitro* expression experiments to ascertain the pathogenicity of a novel mutation (p.Cys655Phe). HEK293 cell lines that stably express each TPO protein (WT or mutant) were established using PiggyBac system. Western blots were performed in triplicate between wild-type, mutant, and control cells (Figure 4(a)). As a result of comparing expression levels with ImageJ, mutant TPO expression normalized against beta-actin was significantly reduced as compared with the wild type. When the wild-type TPO expression level was set to 1, the mutant TPO expression level was 0.274 (Figure 4(b), *P*=0.005 < 0.05). In addition, there was no significant difference between wild type and mutant (*P*=0.990) of RNA expression using real-time PCR (Figure 4(c)). Peroxidase activity in those cell lines was measured using the Amplex Red reaction. The assay of Amplex Red reaction showed that the p.Cys655Phe-TPO had strikingly low peroxidase activity, which was 16.4 ± 8.6% (*P* < 0.001) of WT-TPO (Figure 4(d)).

### 3.4. Immunohistochemistry of TPO

**Figure 5:**
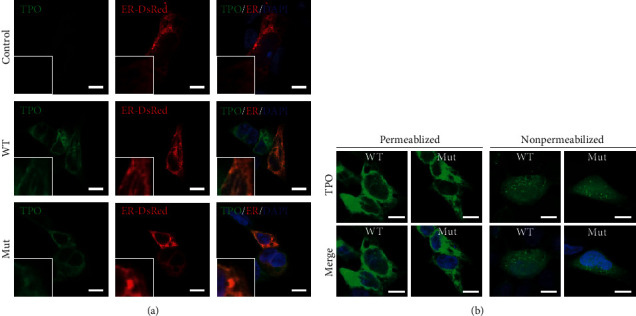
Immunocytochemical analysis of HEK293 cells. Location of wild-type and mutant TPO proteins. To determine the localization of TPO protein, immunofluorescence experiments were performed. Fluorescence was detected in the cells expressing wild-type TPO (WT), p.Cys655Phe (Mut) in both permeabilized and unpermeabilized conditions. (a) Compared under the condition of nonpermeabilized, TPO was localized in the endoplasmic reticulum in both WT and Mut. Cells transfected with the empty vector (control) did not show TPO fluorescence. The TPO is green, the endoplasmic reticulum is red, and the nucleus is purple. (b) WT and Mut were compared under the permeabilized condition (left) and the nonpermeabilized condition (right). There was no significant difference in the localization of TPO between the two types of cells. The presence of TPO was confirmed on the cell membrane surface under nonpermeabilized conditions and on the endoplasmic reticulum under permeabilized conditions. The white bar shows 10 *μ*m.

Immunofluorescence studies were performed to determine the localization of TPO proteins. Immunocytochemical analyses were performed with each cell line expressing wild-type TPO or mutant TPO under both permeabilized and nonpermeabilized conditions (Figures 5(a) and 5(b)). The results indicate that both wild-type TPO and mutant TPO localize to the cell membrane and endoplasmic reticulum.

## 4. Discussion

Using next-generation sequencing, we revealed a novel homozygous TPO mutation (c.1964 G>T, p.Cys655Phe) in the patient with CH. To date, more than 70 TPO mutations have been reported, but only some of them have been assessed in vitro for enzyme activity [17–22]. We demonstrated through in vitro experiments that the TPO activity of the new mutant is significantly lower than that of the WT. Using a molecular graphic tool, we created a three-dimensional image of the molecular structure of this new mutation TPO and confirmed that disulfide bonds disappeared with amino acid substitution.

Proteins inside the endoplasmic reticulum fold correctly by forming disulfide bonds. It has been previously discussed that substitution of cysteine residue disrupted disulfide bridges and induced CH [23]. It is unclear whether TPO mutations prevent intracellular translocation to the plasma membrane surface in thyroid follicular cells. In previous study, a TPO mutation of p.Cys825Arg reported by Zhao et al. has substitutional malfunction of a disulfide-forming cysteine residue in TPO protein [24]. Our experiments revealed that mutant TPO protein (p.Cys655Phe) was abundant not only in the cell membrane but also in the cytoplasm, especially in the endoplasmic reticulum even under nonpermeabilized condition.

## 5. Conclusion

In conclusion, a new TPO homozygous mutation (p.Cys655Phe) was identified in a Japanese family. This single disulfide bond loss mutation reduced TPO activity and caused congenital hypothyroidism without affecting subcellular localization of TPO proteins.

## Data Availability

The data used to support the findings of this study are available from the corresponding author upon request.
